# Effects of Type of Health Insurance Coverage on Colorectal Cancer Survival in Puerto Rico: A Population-Based Study

**DOI:** 10.1371/journal.pone.0096746

**Published:** 2014-05-05

**Authors:** Karen J. Ortiz-Ortiz, Roberto Ramírez-García, Marcia Cruz-Correa, Moraima Y. Ríos-González, Ana Patricia Ortiz

**Affiliations:** 1 Puerto Rico Central Cancer Registry, University of Puerto Rico Comprehensive Cancer Center, San Juan, Puerto Rico; 2 Department of Health Services Administration, Graduate School of Public Health, Medical Sciences Campus, University of Puerto Rico, San Juan, Puerto Rico; 3 University of Puerto Rico Comprehensive Cancer Center, San Juan, Puerto Rico; 4 Cancer Control and Population Sciences Program, University of Puerto Rico Comprehensive Cancer Center, San Juan, Puerto Rico; 5 Department of Biostatistics and Epidemiology, Graduate School of Public Health, Medical Sciences Campus, University of Puerto Rico, San Juan, Puerto Rico; Baylor University Medical Center, United States of America

## Abstract

Colorectal cancer represents a major health problem and an important economic burden in Puerto Rico. In the 1990's, the Commonwealth of Puerto Rico implemented a health care reform through the privatization of the public health system. The goal was to ensure access to health services, eliminate disparities for medically indigent citizens and provide special coverage for high-risk conditions such as cancer. This study estimates the 5-year relative survival rate of colorectal cancer and the relative excess risk of death in Puerto Rico for 2004–2005, by type of health insurance coverage; Government Health Plan vs. Non-Government Health Plan. Colorectal cancer in advanced stages was more common in Government Health Plan patients compared with Non-Government Health Plan patients (44.29% vs. 40.24 had regional extent and 13.58% versus 10.42% had distant involvement, respectively). Government Health Plan patients in the 50–64 (RR = 6.59; CI: 2.85–15.24) and ≥65 (RR = 2.4; CI: 1.72–4.04) age-groups had the greater excess risk of death compared with Non-Government Health Plan patients. Further studies evaluating the interplay of access to health services and the barriers affecting the Government Health Plan population are warranted.

## Introduction

Colorectal cancer (CRC) is the second most commonly diagnosed cancer among males and females, and is the overall leading cause of cancer death in Puerto Rico [1]. Considering that the population of Puerto Rico is aging, we can expect an increase in the burden of this type of cancer. During 1987–2009, the CRC incidence rate increased significantly for both males and females by an annual average of 1.8% and 1.5% respectively [1]. In addition, the CRC mortality rate increased significantly by an annual average of 1.7% in males and 0.2% in females during 1987–2008 [1]. Due to premature mortality, colorectal cancer is the second leading cancer that generates productivity loss in Puerto Rico [2]. Despite the availability of colorectal cancer screening and the efforts to reduce the burden of this type of cancer, it is evident that a considerable health and economic impact persists. According to the Behavioral Risk Factor Surveillance System, screening rates for CRC remain low in Puerto Rico. During 2012, the prevalence of adults aged 50 or more who have ever had a sigmoidoscopy or colonoscopy was 47.2% in Puerto Rico and 67.3% in the United States [3].

It has been shown that for people with cancer, an early diagnosis and appropriate treatment may greatly enhance their chances of survival [4], [5], [6], [7], [8], [9], [10], [11], [12]. Therefore, access to medical care, defined as the timely use of affordable health services meant to achieve the best possible health outcomes, may influence the survival of cancer patients [6],[13]. Beyond access to health services, it has also been argued that health system performance influences the health outcomes of a population [13], [14], [15], [16]. A health system consists of all the organizations, institutions, people, resources, and actions whose primary intent is to promote, restore, or maintain health [15], [16]. Health systems are a means to help achieve health-related goals of each society [13]. For example, it has been shown that better care coordination can improve health system performance in terms of quality of care [17].

Aspects related to delay in cancer diagnosis and treatment could explain, in part, health system performance. Delay in cancer diagnosis and treatment can be categorized into three different types: *patient delay*, from first symptom to first contact with the primary care physicians (PCPs); *doctor delay*, from the first contact with the PCPs to initiation of investigation of cancer-related symptoms; and, *system delay*, from the initiation of investigation of cancer-related symptoms, referral to secondary health care, first visit, and diagnosis/referral to treatment and initiation of the treatment [18], [19]. The health system in Puerto Rico, similar to others, faces a challenge of providing optimal health care with limited financial resources. Understanding these limitations and the policy options available to address them is fundamental to maximize resources and improve the health status of the population.

Prior to 1993, the Commonwealth of Puerto Rico, a territory of the United States, had a regionalized public health system with primary, secondary, tertiary, and supra-tertiary patient care levels. All health care facilities and human resources were owned and financed by the government [20]. Although it operated as a universal health care system, since no citizen was deprived of health services [20], most of its patients were the medically indigent. The system presented limitations and weakness in the provision of health services. In 1993, the Commonwealth of Puerto Rico initiated the implementation of a managed care delivery system with a government health plan (GHP) whose beneficiaries were limited to the Medicaid and Medicare eligible and medically indigent citizens with incomes below 200% of the federal poverty level [21], [22], [23]. Funding for the GHP came from federal (United States) and local funds (Puerto Rico) [22]. The GHP main goal was the integration of healthcare for the medically indigent population to the private health sector. These changes were intended to improve access to health services for the medically indigent, as well as the quality and cost effectiveness of health services [24].

The focus of this investigation is the first period of implementation of the GHP, which lasted until 2009. At the time, the GHP was locally known as *Reforma*. The government employed two strategies to facilitate the transition. First, it contracted the management of access for the care of the medically indigent population to private health insurance companies. This contracting process was based on capitation arrangements. Second, it sold the majority of public healthcare facilities to private investors and providers, including most hospitals and primary care Centers for Diagnosis and Treatment [25]. The entry point to the health care system was then through primary care physicians, who provided services in private offices as individuals or as groups called Independent Practice Associations (IPA). Medically indigent patients were assigned to a primary care physician who determined if their health condition required referrals to specialists, diagnostic tests, or medications [26]. Although this process resulted in a mostly privately operated system, the Puerto Rico Health Department retained responsibility for ensuring the health care of the population, as pursuant to the Constitution of the Commonwealth of Puerto Rico [25].

**Figure 1:**
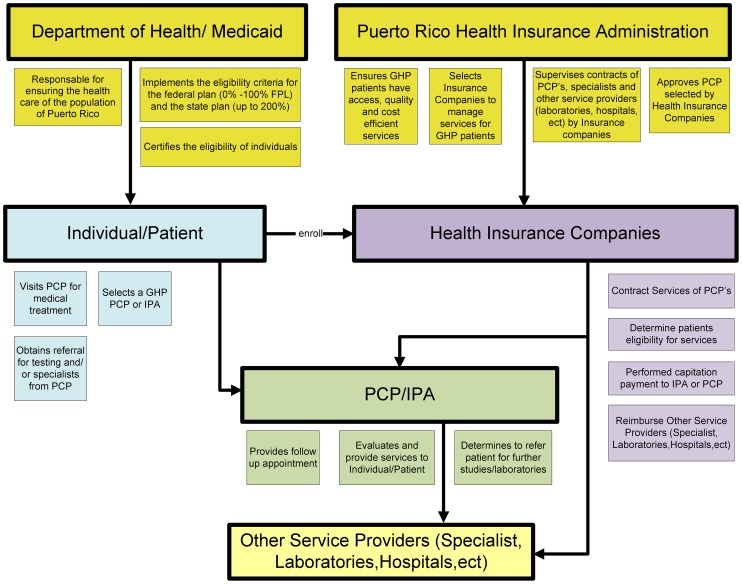
Organizational Process of Puerto Rico Government Health Plan (*Reforma*, 1994–2009). GHP = Government Health Plan FPL = Federal Poverty Level PCP = Primary Care Physician IPA = Independent Practice Associations.

Through the GHP, PCPs assumed the responsibility for coordinating the care of 1.6 million covered lives (40% of the Island's population) [23], [27]. Given that current estimates suggest that only 10% of the population of Puerto Rico does not have health care coverage, approximately 50% of the population is expected to have private coverage [3]. The PCPs contracted their services with insurance companies selected by the government to manage the care of this population through eight regions. All contracting was conducted under the supervision of the Puerto Rico Health Insurance Administration, the administrative body of the GHP (Figure 1). The GHP operated in this manner from 1994–2009.

The goal of the1990's GHP reform was to ensure access to health services and eliminate disparities for medically indigent citizens and provide special coverage for high-risk conditions such as cancer. Among its services, the GHP has a special coverage provision that includes cancer, among others illnesses requiring specialized services. The purpose of this coverage is to facilitate the effective management of the insured population with special health conditions. Cancer coverage under this provision begins upon the confirmation of a cancer diagnosis. A confirmation is obtained through pathology results and/or procedures performed.

Access to health insurance is known to influence the amount and quality of health care received, and thus, the type of insurance may be important to the cancer patient survival [5], [8],[10]. Although socio-demographic factors and the type of insurance status have been shown to be associated to patient's survival, few studies have considered the joint effects of these variables on disease outcomes, particularly among Hispanics. Thus, this study was aimed to estimate the 5-year relative survival rate of CRC in Puerto Rico for 2004–2005 by taking into consideration the impact of the type of health insurance coverage, GHP vs. Non-Government Health Plan (NGHP), on a patient's survival and to estimate the relative excess risk (RER) of death by type of health insurance coverage. In order to assess the impact of the GHP changes in cancer patients, we analyzed the 2004–2005 period, as it has been more than a decade from its implementation. Furthermore, this time period allows for a patient follow-up of five years. This study is particularly relevant as it helps explain the impact of the GHP reform in Puerto Rico, providing important information to formulate a coordinated response for cancer prevention and treatment in Puerto Rico.

## Materials and Methods

### Data sources

Data for patients diagnosed with CRC were provided by the Puerto Rico Central Cancer Registry (PRCCR). The PRCCR is one of the oldest population-based cancer registries in the world, collecting information since 1951 [1]. Since 1997, the PRCCR has been part of the CDC's National Program of Cancer Registry (NPCR) and uses the Surveillance, Epidemiology, and End Results (SEER) Program and the North American Association of Central Cancer Registries (NAACCR) standards for coding data. Notification of cancer has been a legislative requirement for all health facilities in Puerto Rico. PRCCR acquires information from hospitals, outpatient clinics, pathology laboratories, and radiotherapy/chemotherapy sites, throughout the island. PRCCR collects demographic characteristics, date of cancer diagnosis, anatomic cancer site, histology type, method of diagnosis, stage of disease at diagnosis, therapy, and follow-up status. Also, the PRCCR obtains information on vital status and cause of death from all incident cancer cases from the Puerto Rico Department of Health's Statistical Analysis Division [1]. Causes of death are coded and classified according to the International Classification of Diseases, Tenth Edition (ICD-10) [28]. However, PRCCR is a passive follow-up registry maintaining follow-up information by linkages with others databases, like death certificates and health insurances claims databases, among others.

This study included all invasive CRC patients diagnosed with primary cancer from January 2004 to December 2005. Primary site and histology type of invasive CRC were coded according to the International Classification of Diseases for Oncology 3^th^ (ICD-O-3) site codes C180-C209, excluding lymphomas, leukemia, and sarcomas [29]. The cases included in the analysis are only from residents of Puerto Rico. Patients who were diagnosed or treated in Puerto Rico but were residents of another country at the time of the cancer diagnosis were not included. Cases reported to the PRCCR with unknown age, patients 100 years or older, and those identified by death certificate only or at autopsy were excluded for the analysis. In addition, only cases with pathological confirmation were included. Stage at diagnosis was categorized using the Collaborative Staging Derived SEER Summary Stage 2000 that was grouped by “local” (tumor limited to the primary site), “regional” (disease spread beyond the organ of origin either by direct extension or to regional lymph nodes), “distant” (metastasis to distant sites), and “unknown” [30].

The Puerto Rico Health Insurance Administration provided claims related with patients diagnosed with cancer to the PRCCR for 2004–2005 to identify and corroborate GHP patients. After obtaining analytical incident cases of CRC, the PRCCR conducted a probabilistic match to determine which patients were GHP patients. CRC patients in the PRCCR database which were not identified as Puerto Rico Health Insurance Administration beneficiaries were classified as NGHP patients. Similar to the proportion of people who's had GHP in Puerto Rico, 39.15% of CRC patient corresponded to GHP.

### Ethics Statement

The study was reviewed and approved by the institutional review board of the University of Puerto Rico, Medical Sciences Campus, San Juan, Puerto Rico. The study involved a secondary data-analysis of the databases of the PRCCR. All data obtained by the PRCCR from patients are held in strict confidence and patient records de-identified prior to analysis.

### Statistical analysis

In order to evaluate the difference in demographic characteristics and stage at diagnosis a Chi-square or Fisher's exact test were used when appropriate. Five-year maximum relative survival rate was calculated using the cohort method and using a maximum likelihood algorithm in STATA Software [31], [32]. The relative survival rate (which is an alternative to calculating cancer specific mortality), is calculated as the ratio of the observed survival to the expected survival for a group of people in a general population that is similar to that of the patient group with respect to race, sex, age, and calendar period of observation [8], [31], [32], [33], [34]. Thus, the relative survival rate of Puerto Rico was calculated using the expected survival of the Puerto Rican population [35]. Expected survival was calculated based on decennial life table for the Puerto Rico population, which takes into account the population distribution of age, sex, and calendar year [35]. Analyses were performed using Stata/SE version 11.2 statistical software (Stata Corp., LP., College Station, TX). Relative survival rate analysis is the method most commonly used to describe survival of cancer patients in population based studies. Because all deaths are included, the death certificate is not required, only the fact and date of death. This approach avoids errors of misclassification (whether the death was due to cancer) that can arise with cancer-specific survival [31], [34]. The analysis of the survival data was obtained from the PRCCR, a passive follow-up registry, that has the potential to produce unknown random losses, which would affect estimates of survival [33]. Therefore, any registered cancer patient whose death has not been notified to the PRCCR is considered to be surviving [33], [34].

In addition to calculating survival, we modelled the effect of health insurance coverage type categories on relative cancer survival rate, while stratifying by stage and age group; and adjusting for sex and year of follow-up, using a Poisson regression model for excess deaths. The reference insurance plan group in the RER of death estimation was NGHP. The likelihood ratio test statistic was used to assess the significance of interaction terms.

## Results

A total of 2,728 CRC patients were eligible for analyses. The distribution of cases by type of health insurance coverage is given in Table 1. A significant (p<0.05) difference in the proportion of CRC patients by age was observed by health insurance coverage type. Although a similar proportion of CRC patients aged <50 years was observed in the GHP (11.52%) and NGHP (9.10%) groups, a higher proportion of GHP patients were in the 50–64 year age-group (38.76% versus 28.61% in the NGHP group) whereas more NGHP patients were in the ≥65 years age-group (62.29% vs. 49.72% in the GHP group). In terms of sex distribution, NGHP CRC patients had a higher proportion of males (53.19%) compared with GHP (48.97%). There were significant (p<0.05) differences on stage at diagnosis between the two groups, with 46.33% of CRC cases diagnosed in the group of NGHP having localized disease as compared to 35.21% in the group of GHP. Among NGHP 40.24% had regional extent, 10.42% had distant involvement, and 3.01% had unknown stage. Regarding GHP patients, 44.29% had regional extent, 13.58% had distant involvement, and 6.93% had unknown stage.

**Table 1:** Demographic Variables for Colorectal Cancer Cases in Puerto Rico, by Type of Health Insurance Coverage.

Category	Subcategory	Non-Government Health Plan N (%)	Government Health Plan N (%)	Total (%)	p-value
All		1,660 (60.85)	1,068 (39.15)	2,728 (100.0)	
Median Age&		68	64	67	<0.001
Age Group**	<50 y	151 (9.10)	123 (11.52)	274 (10.04)	<0.001
	50–64 y	475 (28.61)	414 (38.76)	889 (32.59)	
	>64 y	1,034 (62.29)	531 (49.72)	1,565 (57.37)	
Sex **	Male	883 (53.19)	523 (48.97)	1,406 (51.54)	0.031
	Female	777 (46.81)	545 (51.03)	1,322 (48.46)	
Stage at Diagnosis**	Localized	769 (46.33)	376 (35.21)	1,145 (41.97)	<0.001
	Regional	668 (40.24)	473 (44.29)	1,141 (41.83)	
	Distant	173 (10.42)	145 (13.58)	318 (11.66)	
	Unknown	50 (3.01)	74 (6.93)	124 (4.55)	

** χ^2^ Test significant at p<0.05.

& Wilcoxon statistics.

Table 2 presents overall one-year, three-year, and five-year relative survival of CRC patients in Puerto Rico; stratified by health insurance, sex, age at diagnosis, and stage at diagnosis. The overall relative survival of CRC patients in Puerto Rico after five years of diagnosis was 62.31% (95% CI = 60.10%, 64.48%). The overall (all stages) five-year relative survival was notably higher for NGHP patients (70.94%, 95% CI: 68.10%, 73.67%) compared with GHP patients (49.11%, 95% CI: 45.68%, 52.49%) (p<0.05). Overall, patients younger than 50 years have a better survival in the first year compared to other age groups. However, the five-year survival of these younger CRC patients was worse than the survival of patients in the older age groups (50–64 years and ≥65 years). CRC patients also had different patterns of survival depending on stage at diagnoses. Patients with localized disease at the time of diagnosis had a longer survival than those with regional, distant or unknown disease at diagnosis. This pattern was also observed when data was stratified by age group (data not shown).

**Table 2:** Relative Survival (1, 3 and 5 Years) of Colorectal Cancer Cases in Puerto Rico*.

Category	Subcategory	N	1-year survival (95% CI)	3-year survival (95% CI)	5 year- survival (95% CI)
Overall		2,728	81.94 (80.33, 83.46)	68.38 (66.36, 70.34)	62.31 (60.1, 64.48)
Health Insurance	Non-Government Health Plan	1,660	84.01 (81.98, 85.86)	74.66 (72.12, 77.08)	70.94 (68.10, 73.67)
	Government Health Plan	1,068	78.75 (76.02, 81.24)	58.73 (55.43, 61.91)	49.11 (45.68, 52.49)
Sex	Male	1,406	82.25 (79.95, 84.35)	67.03 (64.12, 69.82)	60.75 (57.57, 63.85)
	Female	1,322	81.62 (79.29, 83.75)	69.78 (66.93, 72.48)	63.92 (60.82, 66.91)
Age at cancer diagnosis	<50 y	274	86.02 (81.28, 89.66)	70.00 (64.11, 75.14)	56.79 (50.60, 62.53)
	50–64 y	889	85.86 (83.32, 88.07)	73.02 (69.81, 75.98)	65.75 (62.28, 69.03)
	>64 y	1,565	78.88 (76.55, 81.05)	65.33 (62.44, 68.12)	61.59 (58.37, 64.75)
Stage at diagnosis	Localized	1,145	90.98 (88.91, 92.76)	83.96 (81.16, 86.52)	80.80 (77.58, 83.82)
	Regional	1,141	85.05 (82.64, 87.21)	69.18 (66.02, 72.18)	61.41 (57.95, 64.77)
	Distant	318	52.69 (46.90, 58.17)	25.16 (20.31, 30.31)	14.01 (10.22, 18.43)
	Unknown	125	44.68 (35.49, 53.55)	27.98 (19.89, 36.78)	23.93 (16.17, 32.77)

*Relative survival and 95% confidence intervals (CI) are given as percentage.

The likelihood ratio statistical test showed a significant interaction between the main predictor variable (health insurance coverage type) and both the covariate age group at diagnosis (p<0.05) and stage at diagnosis (p<0.05). Thus, the Poisson model was stratified by age and stage at diagnosis.

**Figure 2:**
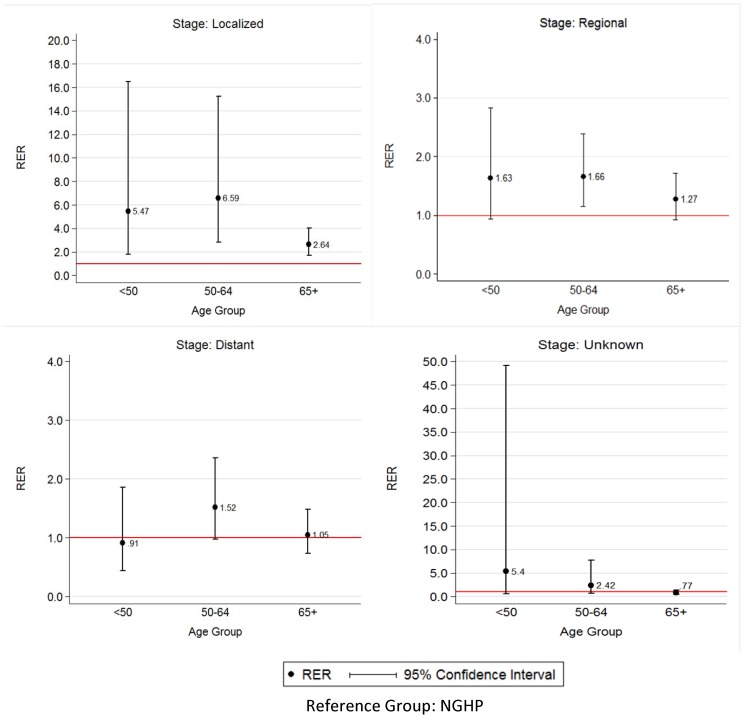
Relative Excess Risk of Death for CRC Cases with GHP compared to NGHP, by Stage at Diagnosis and Age-Group. RER estimated by Poisson regression. Model control for sex and length of follow-up and stratified by stage at diagnosis and age at diagnosis.

RER of death for colorectal cancer are illustrated in Figure 2. Among colorectal cancer patients with localized stage, significant differences in the risk of death were observed between GHP and NGHP for all age groups. However, GHP patients in the 50–64 age groups had the greater excess risk of death within five years of diagnosis, with more than 6 times excess risk of death compared with NGHP (RER = 6.59; 95% CI: 2.85–15.24).

Among CRC patients with regional stage, a significant higher risk of death was observed for GHP patients ages 50–64 years, these having 66% (RER = 1.66, 95% CI: 1.14, 2.39) higher risk of death within five years of diagnosis (p<0.05). Nonetheless, GHP patients with ages younger than 50 years had marginal significant differences in the risk of death compared with the NGHP (p = 0.08). On the other hand, no statistical difference (p>0.05) was observed between GHP and NGHP patients aged 65 years or older.

Furthermore, among CRC patients with distant stage, only a marginal difference in the RER of death was observed between GHP and NGHP patients for the age group 50–64 years. In this group the GHP patients had 52% higher risk of death (RER = 1.52, 95% CI = 0.98, 2.36) within five years compared with the NGHP patients. Meanwhile, among CRC patients with unknown stage, although the estimates have a similar pattern, no significant difference was observed between GHP and NGHP patients for all age groups.

## Discussion

The analysis of health outcomes across different sectors of society provides an effective way to determine the impact of health care policies and measure the effectiveness of its implementations. Providing policy makers with evidence-based research allows for the corresponding corrective actions to take place and health policies to be redirected, in order to meet the population needs. It has been stated that cancer survival is a key measure of health system performance [36].

This study confirms that CRC patients who had GHP are diagnosed at an advanced stage and had a lower relative survival compared with NGHP patients, which highlights a health disparity that warrants further research. These results are relevant as patients diagnosed in later stages tend to have a lower survival rate, because they are more difficult to treat successfully. Another interesting finding was that GHP patients were diagnosed at younger ages (<65 years). Nevertheless, although GHP patients have a younger age at diagnosis, they have a more advance stage at diagnosis than NGHP patients. Thus, future studies should assess the potential risk factor differences between these groups, as well as the social and/or biological determinants of health that may be influencing these patterns.

In addition, GHP patients from all age-groups diagnosed early (localized stage), had a higher risk of dying within five years, compared to NGHP. Furthermore, even though the proportion of CRC patients <50 years was similar in both GHP and NGHP groups, an excess risk of death was observed in this youngest population of GHP patients (particularly those with localized disease) when compared to NGHP. This excess risk of CRC in patients younger than 50 years, in which CRC screening practices are not recommended, supports the potential role of other risk factors in Hispanics that needs to be better understood to develop appropriate intervention strategies, to reduce health disparities within this group. Meanwhile, differences were not that big among GHP and NGHP patients with regional/distant disease, supporting the fact that when disease is advanced, the outcomes are not different between these groups, a result that could be explained by the fact that receiving or not receiving the best treatment does not significantly change the survivorship among individuals with distant/advance stage disease in this population. Again, this highlights the fact that beyond an appropriate screening, GHP patients may have problems with access and quality of health services once diagnosed that impacts localized stage patients more strongly, in which appropriate treatment may have a better result on the disease outcome. This is relevant, as delay in treatment may be a sign of failures in the implementation of the reform of the GHP.

Delay in cancer diagnosis of GHP patients warrant further research to better understand parts of these results. It is important to study the different factors that pertain to the patient delay, such as social conditions, co-morbidities, educational background, knowledge, attitudes, and behavior, among others. Mandelblatt and colleagues [6] stated that strategies to remove economic barriers, such as, improving access to healthcare, are necessary but not sufficient to improve cancer outcomes [6]. In Puerto Rico, it has been found that having a higher level of education is associated with a greater likelihood of having a screening study for colorectal cancer [37]. Behavioral Risk Factor Surveillance System data also evidences that adults aged 50 year or more who report higher income tend to have higher prevalence of ever having a sigmoidoscopy or colonoscopy as compared to those who report less income [3]. Meanwhile, recent studies show that, even with universal access to care, persons with lower socioeconomic status have lower survival rates compared with person with higher socioeconomic status [38], [39], [40], [41].

Despite these factors, the healthcare PCP's delay has been shown as an important factor that could have an influence on the delay of diagnosis [6], [18], [36]. Therefore, another aspect to be studied is doctor delay. Differences in the interval between first visit with a PCP and the referral for diagnostic tests or specialist evaluation, could explain in part why GHP patients are diagnosed at an advanced stage, compared with NGHP. This PCP delay will be especially significant among individuals younger than 50 years of age that do not routinely receive colorectal cancer screening testing. Thus, diagnosis of colorectal cancer will only be prompted by symptoms such as rectal bleeding that will require referral to a specialist. For example, in a recent study, Morse and colleagues [42] found that one barrier to the early diagnosis of oral cancer in Puerto Rico is the lack of knowledge of the healthcare professionals in the detection on the early signs and symptoms of this condition [42]. In the current system, GHP patients are required to visit their PCP for evaluation, treatment, and referral; meanwhile, NGHP patients tend to visit a specialist directly, whose knowledge and expertise extends further than that of the PCP.

Others system barriers that could have an influence on the delay of diagnosis and treatment in cancer patients include organizational and structural factors, reimbursement and financial forces, quality measurement, and regional resources [6]. Managed care capitation system could influence provider behavior and may act as a barrier to cancer care [6]. Morse and colleagues [42] also found that the PCP's in a capitation payment system, such as the GHP, make decisions that can have a personal financial impact on them when considering whether or not to refer a patient [42]. Their study also highlighted inefficiencies associated primarily with the GHP coordination for biopsy referrals and subsequent diagnosis [42].

In addition, another relevant fact evidenced by our study is that GHP patients in the 50–64 age group had the greater excess risk of death within five years of diagnosis, while the other age groups had a lower excess risk. A potential explanation of the difference in the RER of CRC by age group, could be related to the fact that approximately 81% of the population older than 64 years in Puerto Rico are covered by Medicare; either Traditional (Fee for Service) Medicare Advantage or Medicare *Platino* (dual eligible) [43]. Of this group, 53% are dual eligible individuals who are entitled to Medicare Part A and/or Part B and are eligible for some form of Medicaid benefit [43]. Therefore, GHP patients over 64 years of age and dual eligible may have better health coverage.

It is important to highlight that GHP cancer patients have a comprehensive coverage with the Special Coverage provision that seeks to facilitate the effective management of this condition. Nonetheless, disparities in CRC outcomes exist, as has been described in the present study. Factors that should be evaluated in Puerto Rico include the supply of specialists (gastroenterologists) who accept the GHP and the difficulty to obtain the referral to a gastroenterologist among GHP patients, as these could help explain the disparity observed in our study between GHP and NGHP patients. Another aspect that needs further investigation is whether there is a difference in the quality of care between these groups. For example, if the established treatment guidelines are being followed, and if limitations regarding access to oncologic therapy, chemotherapeutic agents or surveillance exist. The fragmentation of services and poor coordination could be playing an important role.

The current analysis is focused on the study period of 2004–2005, in which the first period of implementation of the GHP (*Reforma*) was active. Thus, our results may not reflect current survival outcomes of CRC patients in Puerto Rico, as the government in 2010 implemented additional changes to the GHP, known locally from this date forward as *Mi Salud*. With this implementation, GHP (*Mi Salud*) brought a set of new changes to improve the delivery of health care services to the Medicaid population in Puerto Rico [43]. The current GHP has two major objectives: to integrate physical and mental health services in a coordinated primary care model; and to contract preferred providers in closed-panel networks. The later was done to facilitate patient access to specialized physicians. The specialty services within the Preferred Provider Network are provided without the need for referral [43]. In this program, insurance companies continue to provide managed care services and are responsible for contracting the preferred providers. Thus, it will be important to evaluate if the 2010 changes to the GHP, including access to specialist physicians without referral, will help decrease CRC disparities documented by our study.

A limitation of this investigation was that we could not assess other variables of interest, like socioeconomic status, co-morbidities, and treatment variables, among others that may affect CRC survival. However, factors such as co-morbidities should be minimized in the younger cohort (<50 years old). Another limitation is that the percentage of the NGHP group without insurance is unknown. Consequently, this could lead to an underestimation of the survival for this group. In addition, the use of maximum survival may result in an overestimate of the true survival rate [33]. However, this would be true for all observations (NGHP and GHP).

## Conclusions

The goal of the changes in the GHP in the 1990's was to ensure access to health services and eliminate disparities for medically indigent citizens and was intended to improve the quality and cost effectiveness of health services in Puerto Rico [24]. Nevertheless, health spending in Puerto Rico is approximately 20% of the gross domestic product, making it one of the most expensive in the world [44]. The total health spending amounted to $12.8 billion annually (2011), of which $7.2 billion corresponded to the public sector; the local government spends $2.33 billion and the federal government funds the remaining $4.9 billion [44]. The notion that spending more on health will as a consequence improve the overall performance is not necessarily correct. The observed survival disparities in patients with CRC in Puerto Rico could be an indicative that the goals of the reform of the GHP have not been achieved entirely. Therefore it is important to evaluate the interplay of access to health services and the barriers affecting the GHP population, which warrants further studies in this area.

Access to health services by itself is not a guarantee of better health and better quality of life for a society. GHP's population may have some characteristics that must be taken into account when providing health services. For example, GHP patients may have differences in lifestyles, co-morbidities, family and social support, and socio-demographics factors that may influence the access to health services, as well as geographical challenges that may limit transportation and nearby access to certain specialists. These characteristics require creating an adequate health model to provide effective and timely health services to this population. It is necessary to study different health care delivery approaches and investigate what model of health care is the most cost effective for this population. A key issue is to build better polices and institutions with measurable results and principles of accountability.

Health systems are complex systems; hence their results are the consequences of many multi-casual determinants. It is therefore essential to understand the different determinants that affect the health in a society. This way, policymakers can implement effective public policies and ensure suitable allocation of funds to address various health problems in society.
